# VesselMetaKAN: vessel-guided meta-learned interpretable classification for diabetic retinopathy grading

**DOI:** 10.3389/fmed.2026.1846788

**Published:** 2026-07-16

**Authors:** TianQi Yang, GuoYong Chen, Lu Liu

**Affiliations:** 1 The First Affiliated Hospital of Guangxi Medical University, Nanning, Guangxi, China; 2 The Second Affiliated Hospital of Guangxi Medical University, Nanning, Guangxi, China; 3 GuangXi Health Science College, Nanning, Guangxi, China

**Keywords:** deep learning, diabetic retinopathy, fundus imaging, interpretable classification, Kolmogorov-Arnold networks, meta-learning, public health screening, vessel segmentation

## Abstract

**Introduction:**

Diabetic retinopathy (DR) is a leading cause of preventable blindness worldwide, affecting approximately 93 million people globally. Accurate automated grading is critical for large-scale public health screening, yet existing deep learning methods face challenges related to structure-coupled sparse evidence and domain shift across imaging conditions.

**Methods:**

We propose VesselMetaKAN, a two-stage framework integrating explicit vessel guidance with meta-learning. Stage 1 employs GMF-SwinUnet for topology-aware vessel segmentation using Frangi-guided attention fusion, reliability gating, and clDice loss. Stage 2 employs KAN-MAML, combining Kolmogorov-Arnold networks with radial basis function expansions as an interpretable decision head and Reptile-style meta-learning over augmentation-defined tasks. Vessel probability maps from Stage 1 guide Stage 2 classification through vessel-gated feature fusion.

**Results:**

On APTOS 2019, VesselMetaKAN achieved 74.9 ± 0.4% accuracy, 58.7 ± 0.6% macro-F1, and 0.838 ± 0.005 QWK over five runs. The best single run reached 75.44% accuracy, 59.44% macro-F1, and 0.843 QWK, outperforming EfficientNet-B4 on the principal grading metrics with paired bootstrap significance (*p* < 0.05). Ablation studies confirmed the synergistic contributions of vessel guidance, KAN-based interpretability, and meta-learning.

**Discussion:**

VesselMetaKAN provides a principled, structure-aware, and interpretable solution for robust DR grading in public health screening programs.

## 1 Introduction

Diabetic retinopathy (DR) is a microvascular complication of diabetes mellitus and a leading cause of preventable blindness worldwide, affecting approximately 93 million people globally (1). Early detection and timely intervention are critical for preventing vision loss, yet manual screening by ophthalmologists is time-consuming, expensive, and subject to inter-observer variability (2, 3). The World Health Organization estimates that the number of people with diabetes will rise to 642 million by 2040, creating an urgent need for automated, accurate, and scalable DR screening systems (4). Recent reviews summarize the rapid progress and remaining challenges of deep learning for fundus-based DR detection (5, 6), while scoping reviews of deep learning for large-scale medical imaging screening underscore the importance of robustness and clinical deployment (7). Fundus photography provides a non-invasive imaging modality for DR assessment, where pathological changes such as microaneurysms, hemorrhages, exudates, and neovascularization manifest as visual abnormalities in retinal images (8). However, the subtle and diverse nature of these lesions, combined with significant inter-image variability in illumination, contrast, and image quality, poses substantial challenges for automated grading systems.

Traditional DR grading methods rely on hand-crafted features such as lesion detection, vessel segmentation, and morphological analysis, which require domain expertise and fail to capture complex patterns (9, 10). The advent of deep learning has revolutionized medical image analysis, with convolutional neural networks (CNNs) achieving remarkable success in DR classification (2, 8). Bibliometric analyses likewise document rapid expansion of neural-network-based methods across biomedical sensing and interface applications (11). Recent approaches leverage advanced architectures such as ResNet (12), EfficientNet (13), Vision Transformers (14), and Swin Transformers (15) to learn hierarchical representations directly from fundus images. Attention mechanisms (16–18) and multi-scale or multi-view fusion strategies (19–22) further improve performance by focusing on diagnostically relevant regions. Despite these advances, existing methods treat DR grading as a generic image classification task, ignoring the critical role of retinal vascular structures in disease pathogenesis and diagnosis. Pathological changes in DR are intrinsically coupled with vascular abnormalities—microaneurysms occur at capillary walls, hemorrhages follow vessel trajectories, and neovascularization represents abnormal vessel growth (23).

This fundamental limitation gives rise to two critical challenges. *Challenge 1: Structure-Coupled Sparse Evidence*. Diagnostic features in DR are tightly coupled with vascular structures, which occupy only 10–15% of the fundus image area (24). Fine vessels, particularly capillaries where early pathological changes occur, exhibit low contrast and are easily obscured by noise and image artifacts (25, 26). Without explicit vessel localization, deep networks must simultaneously learn to detect vessels and identify pathological changes, leading to inefficient feature learning. Moreover, the topology of the vascular network is critical for distinguishing normal variations from pathological neovascularization, yet standard pixel-wise losses do not enforce topological constraints (27). *Challenge 2: Domain Shift and Imaging Variability*. Fundus images exhibit substantial variability due to differences in camera models, lighting conditions, patient demographics, and acquisition protocols (18, 28). Models trained on data from one source often fail to generalize to new imaging conditions (29). Meta-learning, which explicitly optimizes for fast adaptation across related tasks, offers a principled solution but has been underexplored in medical imaging (30, 31).

To address these challenges, we propose VesselMetaKAN, a novel framework that integrates explicit vessel guidance, topology-aware segmentation, interpretable classification, and meta-learning for robust DR grading. Our approach consists of four synergistic components: (1) GMF-SwinUnet for vessel segmentation, employing Frangi filtering (32) to generate vessel probability maps as structural priors, Frangi-Guided Attention Fusion (F-GAF) modules, reliability gating, and a topology-aware clDice loss (27); (2) vessel-gated feature fusion, where vessel probability maps *P*_*v*_ explicitly modulate multi-scale features extracted by an EfficientNetV2-S backbone (33); (3) a KAN decision head (34) with radial basis function (RBF) expansions for interpretable non-linear classification; and (4) Reptile-style meta-learning (31) over augmentation-defined tasks to learn robust initializations.

The main contributions of this study are as follows: (i) VesselMetaKAN, the first framework to integrate explicit vessel segmentation, topology-aware losses, interpretable KAN-based classification, and meta-learning for DR grading; (ii) GMF-SwinUnet with Frangi-guided attention, reliability gating, and clDice loss for topology-aware vessel segmentation; (iii) a vessel-gated feature fusion mechanism that explicitly leverages vessel probability maps to modulate classification features; (iv) demonstration that KAN decision heads with RBF basis expansions provide superior interpretability and performance compared to standard MLPs; and (v) strong performance on APTOS 2019 (mean over five runs: 74.9 ± 0.4% accuracy, 58.7 ± 0.6% macro-F1, 0.838 ± 0.005 QWK; best run: 75.44%, 59.44%, and 0.843 QWK).

## 2 Related work

### 2.1 Diabetic retinopathy grading

Early automated DR grading systems relied on hand-crafted features and classical machine learning. Niemeijer et al. (9) proposed lesion-specific detectors for microaneurysms, exudates, and hemorrhages, combining outputs via ensemble classifiers. Sopharak et al. (10) used mathematical morphology for exudate detection. While interpretable, these methods require extensive domain knowledge and fail to capture complex spatial patterns.

The advent of deep learning revolutionized DR grading. Gulshan et al. (2) trained Inception-v3 on 128,000 fundus images, achieving ophthalmologist-level sensitivity and specificity. Abràmoff et al. (3) later demonstrated improved referable DR detection on publicly available datasets through deep learning integration. Gargeya and Leng (8) demonstrated that ResNet-50 could achieve 94% sensitivity with minimal pre-processing. Wang et al. (17) introduced Zoom-in-Net, which progressively focuses on lesion-rich regions via attention mechanisms. Li et al. (16) proposed CABNet, combining channel and spatial attention to highlight diagnostically relevant features. Zhou et al. (19) explored collaborative learning between segmentation and classification tasks. Recent hybrid CNN–Transformer architectures (35) and improved object detectors (36) further expand the design space for medical image analysis. Recent works leverage Vision Transformers (14) and Swin Transformers (15) for global context modeling. Despite impressive performance, these methods treat DR grading as generic image classification, ignoring the critical role of vascular structures.

### 2.2 Retinal vessel segmentation

Classical vessel segmentation methods include matched filtering (37), morphological operations, and Frangi filtering (32), which detects tubular structures via Hessian matrix eigenvalue analysis. Partial differential equation (PDE) formulations provide an alternative variational approach to joint segmentation and denoising in medical imaging (38, 39). Deep learning approaches have achieved state-of-the-art performance: U-Net (40) introduced skip connections for precise localization; Attention U-Net (41) used attention gates to suppress irrelevant features; Swin-Unet (42) replaced convolutional layers with Swin Transformer blocks for long-range dependencies. Maninis et al. (43) proposed deep retinal image understanding for joint analysis of fundus structures. Shit et al. (27) introduced clDice loss, which explicitly penalizes disconnections by comparing predicted and ground-truth skeletons, significantly improving vascular connectivity. Existing vessel segmentation methods are typically trained independently and not integrated with downstream classification tasks.

### 2.3 Kolmogorov-Arnold networks and meta-learning

Traditional multilayer perceptrons (MLPs) use fixed activation functions, limiting expressiveness. Liu et al. (34) proposed KAN, inspired by the Kolmogorov–Arnold representation theorem (44), replacing weight matrices with learnable basis function expansions. This enhances interpretability as decision boundaries can be visualized via basis function responses. KAN has shown superior performance in scientific computing but remains underexplored in medical imaging.

Meta-learning optimizes for fast adaptation across related tasks. MAML (30) learns an initialization that requires few gradient steps to adapt to new tasks. Reptile (31) simplifies MAML by using first-order gradients, reducing computational cost. While meta-learning has been applied to few-shot classification and domain adaptation (29, 45), its application to medical imaging robustness is limited. To the best of our knowledge, VesselMetaKAN is the first work to integrate explicit vessel segmentation, topology-aware losses, interpretable KAN-based classification, and meta-learning for DR grading.

## 3 Materials and methods

### 3.1 Framework overview

**Figure 1:**
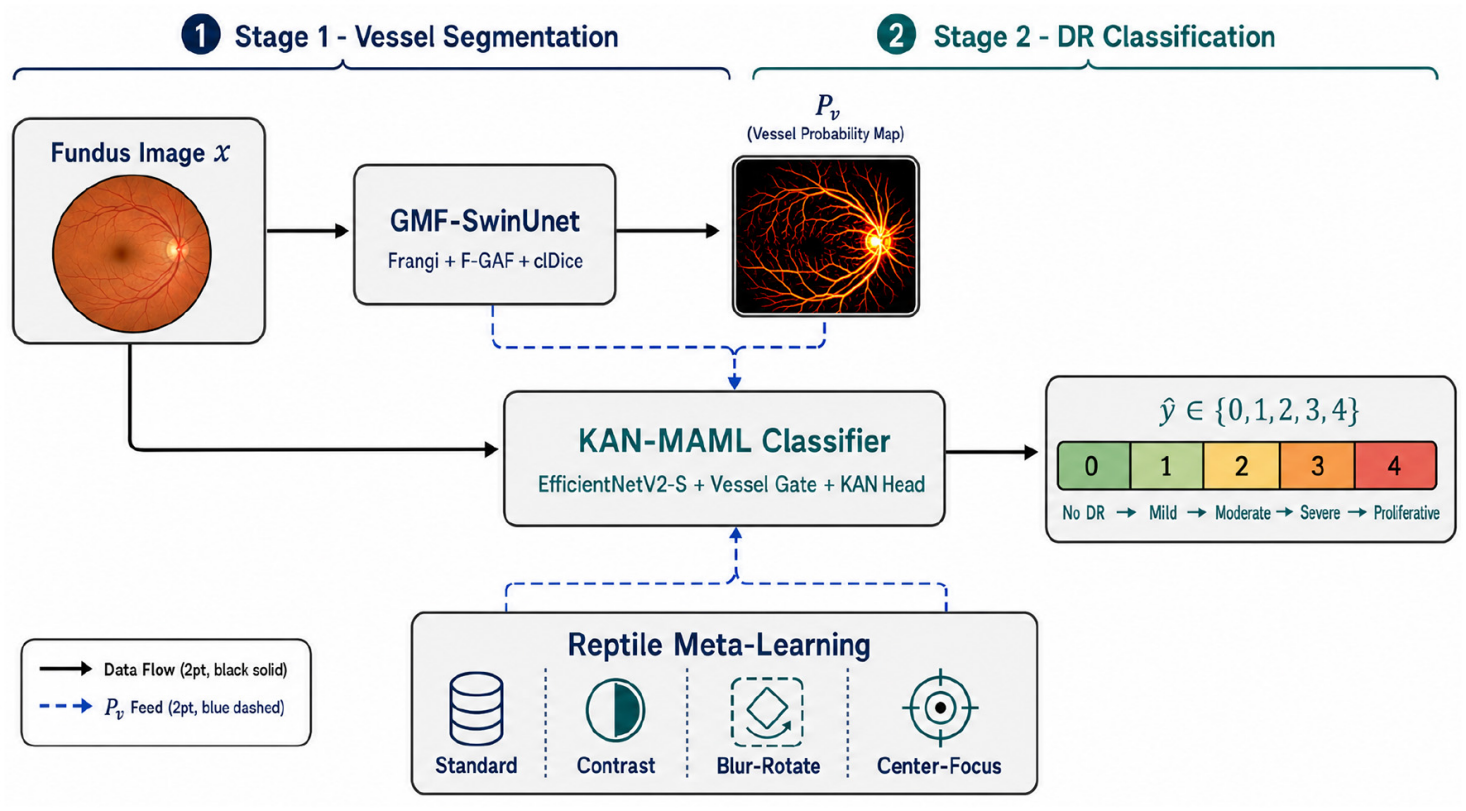
Overall architecture of VesselMetaKAN. Stage 1 (GMF-SwinUnet) produces vessel probability map *P*_*v*_; Stage 2 (KAN-MAML) uses *P*_*v*_ for vessel-gated fusion and meta-learning to predict DR grade ŷ.

Figure 1 illustrates the overall architecture of VesselMetaKAN. The framework consists of two main stages: (1) *Vessel Segmentation* via GMF-SwinUnet, which takes a fundus image **x** ∈ ℝ^*H* × *W* × 3^ and outputs a vessel probability map $P_{v}\in {\left[0,1\right]}^{H\times W}$; (2) *DR Classification* via KAN-MAML, which takes **x** and *P*_*v*_ as inputs and predicts the DR grade ŷ ∈ {0, 1, 2, 3, 4}. The vessel probability map *P*_*v*_ explicitly guides classification through vessel-gated feature fusion at multiple scales. Meta-learning over augmentation-defined tasks learns robust initializations that rapidly adapt to diverse imaging conditions.

### 3.2 GMF-SwinUnet: topology-aware vessel segmentation

**Figure 2:**
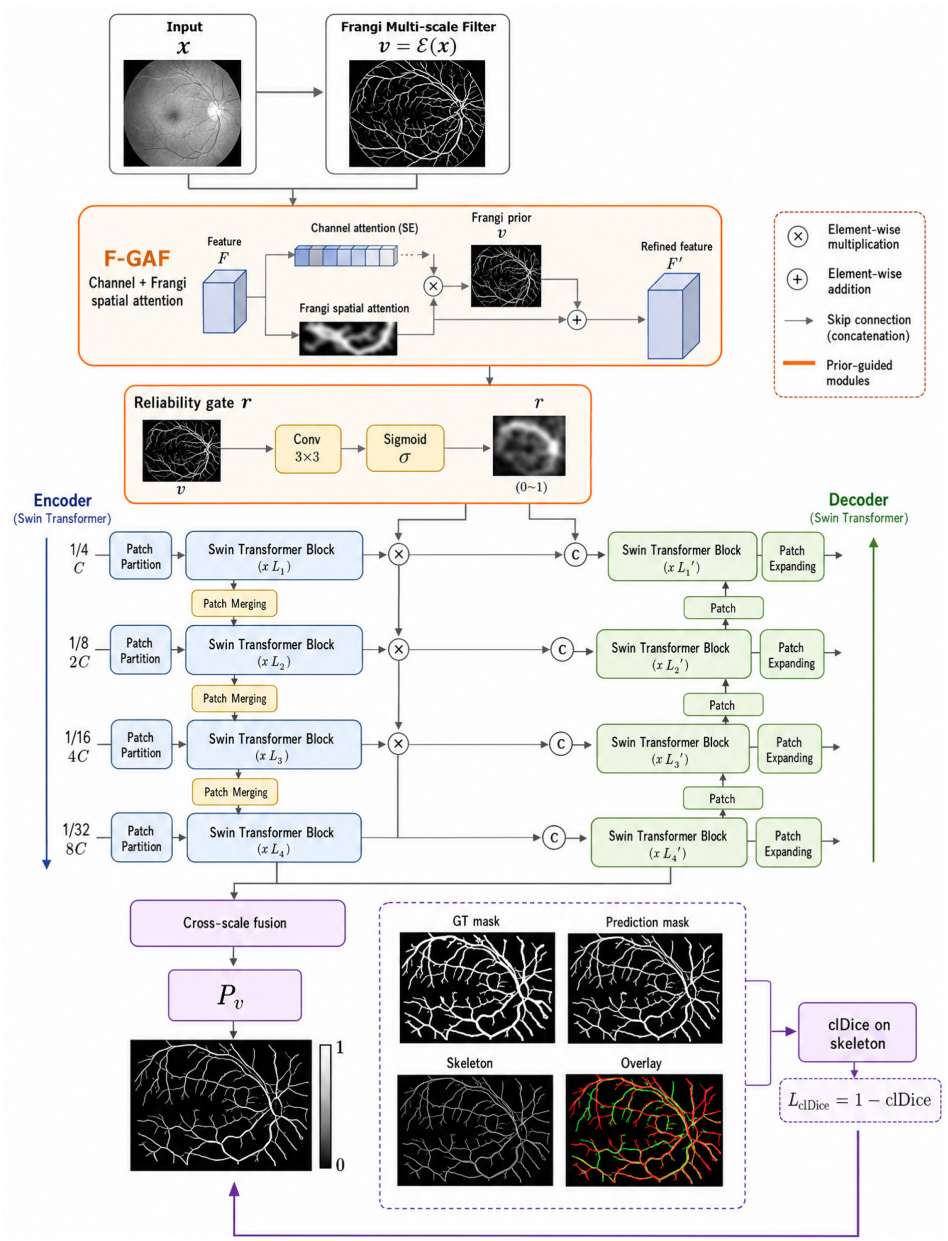
Stage 1 (GMF-SwinUnet): Frangi preprocessing → encoder with F-GAF and reliability gating → decoder with cross-scale fusion → vessel probability map *P*_*v*_.

Figure 2 illustrates the detailed architecture of Stage 1, GMF-SwinUnet, including Frangi preprocessing, F-GAF, reliability gating, cross-scale fusion, and vessel probability map generation.

#### 3.2.1 Frangi filter pre-processing

We employ Frangi filtering (32) to generate vessel probability maps as structural priors. For a grayscale fundus image **x**, we compute the Hessian matrix **H**(**x**; σ) at scale σ as shown in Equation 1. The vesselness measure is defined in Equation 2.


$$\begin{array}{l} \text{H}\left(\text{x};\sigma \right)=\left[\begin{array}{cc} I_{xx} & I_{xy}\\ I_{xy} & I_{yy} \end{array}\right], \end{array}$$ (1)

where *I*_*xx*_, *I*_*xy*_, and *I*_*yy*_ are second-order derivatives obtained via Gaussian convolution. The eigenvalues λ_1_*andλ*_2_ (with |λ_1_| ≤ |λ_2_|) characterize local structure: for vessels, |λ_1_| ≪ |λ_2_| and λ_2_ < 0. The vesselness measure is as follows:


$$v_{\sigma }(x)=\{\begin{array}{ll} 0, & {\text{if λ}}_{2}>0\\ \exp\text{​}\left(-\frac{R_{b}^{2}}{2\beta^{2}}\right)\text{​}\left(1-\exp\text{​}\left(-\frac{S^{2}}{2c^{2}}\right)\right), & \text{otherwise} \end{array}$$ (2)

where *R*_*b*_ = |λ_1_|/|λ_2_|, $S=\sqrt{{\text{λ}}_{1}^{2}+{\text{λ}}_{2}^{2}}$, and β*andc* are tunable parameters. Multi-scale vesselness is $\text{v}\left(\text{x}\right)=\underset{\sigma \in \text{Σ}}{\max}{\text{v}}_{\sigma }\left(\text{x}\right)$, where Σ = {0.5, 1.0, 1.5, 2.0, 2.5, 3.0}.

#### 3.2.2 F-GAF: Frangi-guided attention fusion

The F-GAF module explicitly modulates encoder features **F** ∈ ℝ^*B* × *C* × *H* × *W*^ using the Frangi vesselness map **v** via two parallel branches. The *channel attention branch* computes **w**_*c*_ = σ(Conv_1 × 1_(GAP(**F**))) and produces **F**_*c*_ = **F**⊙**w**_*c*_, where GAP(·) is global average pooling and ⊙ denotes element-wise multiplication. The *The Frangi spatial attention branch* is computed as shown in Equation 3.


$$\begin{array}{l} {\text{F}}_{f}=\text{F}⊙\left(1+\gamma \cdot {\text{v}}_{spatial}\right), \end{array}$$ (3)

where **v**_*spatial*_ = Resize(**v**, *H, W*) and γ is a learnable scalar. The final output is **F**_*out*_ = Conv_3 × 3_(Concat([**F**_*c*_, **F**_*f*_]))+**F**.

#### 3.2.3 Reliability gating

Frangi filtering may produce false positives in noisy or low-quality regions. We introduce a reliability gate **r** ∈ [0, 1]^*H* × *W*^. The reliability gate and gated feature modulation are defined in Equations 4 and 5.


$$\begin{array}{l} \text{r}=\sigma \left({\text{Conv}}_{3\times 3}\left(\text{Concat}\left(\left[\text{F},{\text{v}}_{spatial}\right]\right)\right)\right), \end{array}$$ (4)

$$\begin{array}{l} {\text{F}}_{f}=\text{F}⊙\left(1+\gamma \cdot \text{r}⊙{\text{v}}_{spatial}\right). \end{array}$$ (5)

The reliability gate learns to suppress Frangi responses in unreliable regions while enhancing them in high-confidence vessel areas.

#### 3.2.4 Topology-aware segmentation loss

Standard Dice loss optimizes pixel-wise overlap but ignores topology. We combine clDice (27) with DiceBCE loss. The combined segmentation loss is defined in Equation 6, and the topology precision and recall used for clDice are defined in Equation 7.


$$\begin{array}{l} L_{seg}={\text{λ}}_{cl}L_{clDice}+{\text{λ}}_{db}L_{DiceBCE}, \end{array}$$ (6)

where λ_*cl*_ = 0.3 and λ_*db*_ = 0.7. The clDice loss computes topology precision (TP) and recall (TR) on skeletonized predictions:


$$\begin{array}{l} \text{TP}=\frac{\sum \left(\text{Skel}\left(\hat{M}\right)⊙M\right)}{\sum \text{Skel}\left(\hat{M}\right)+\epsilon },\;\text{TR}=\frac{\sum \left(\text{Skel}\left(M\right)⊙\hat{M}\right)}{\sum \text{Skel}\left(M\right)+\epsilon }, \end{array}$$ (7)

and $L_{clDice}=1-2\text{TP}\cdot \text{TR}/\left(\text{TP}+\text{TR}\right)$. The DiceBCE loss is $L_{DiceBCE}=0.5L_{Dice}+0.5L_{BCE}$.

### 3.3 KAN-MAML: interpretable meta-learned classification

**Figure 3:**
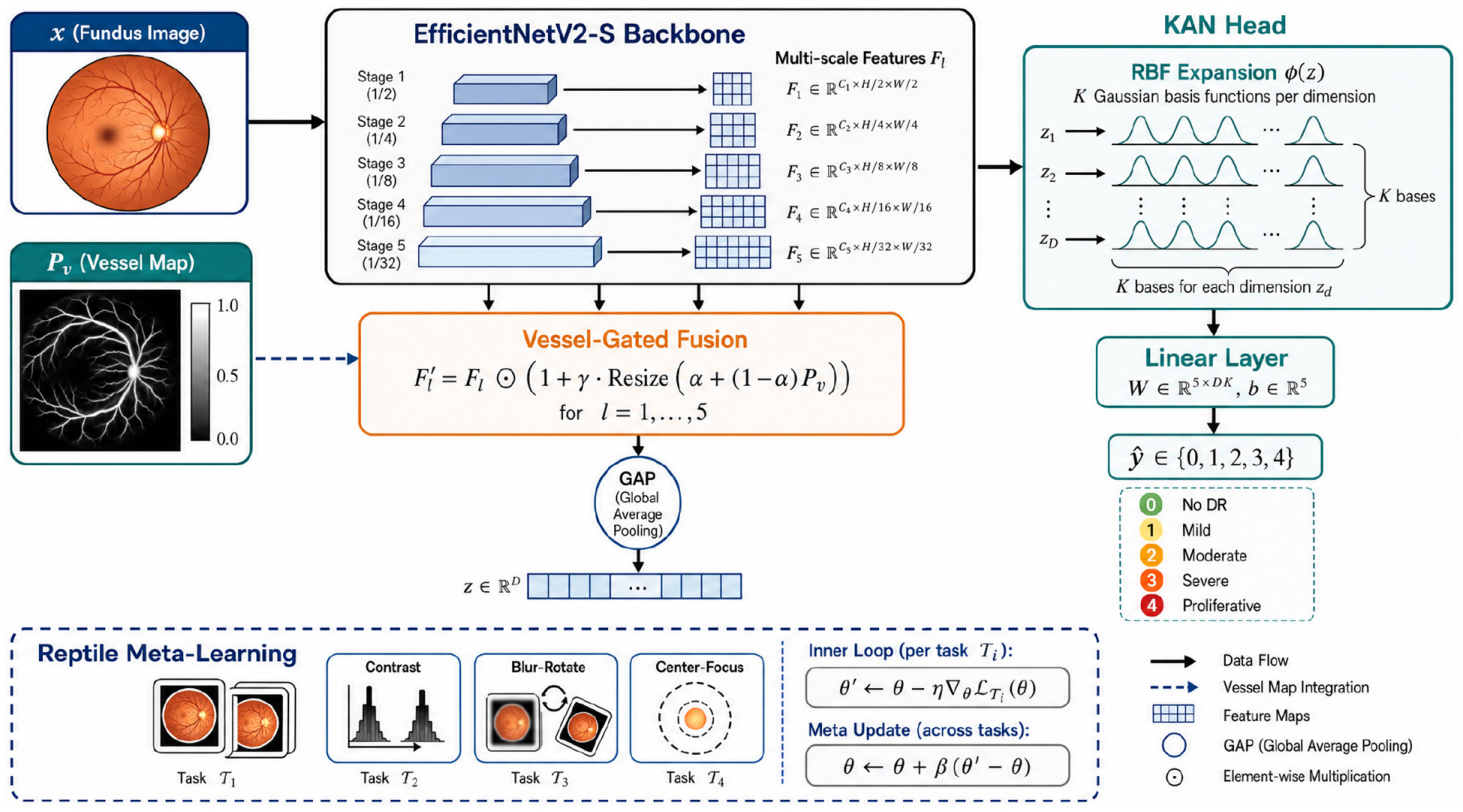
Stage 2 (KAN-MAML): Input **x** and *P*_*v*_ → EfficientNetV2-S backbone with vessel-gated fusion → global feature **z** → KAN decision head (RBF expansion) → DR grade ŷ.

Figure 3 shows the Stage 2 KAN-MAML classification framework, including the EfficientNetV2-S backbone, vessel-gated fusion, KAN decision head, and Reptile meta-learning module.

#### 3.3.1 EfficientNetV2-S backbone and vessel-gated Fusion

We use EfficientNetV2-S (33) as the feature extractor, producing multi-scale features ${\left\{{\text{F}}_{l}\right\}}_{l=1}^{L}$ and a global feature vector $\text{z}=\text{GAP}\left({\text{F}}_{L}\right)\in {\mathbb{R}}^{1280}$. Following multi-view fusion principles (20–22), the vessel probability map *P*_*v*_ modulates multi-scale features via. The vessel-gated feature fusion is defined in Equation 8.


$$\begin{array}{l} {\text{F}}_{l}^{\prime }={\text{F}}_{l}⊙\left(1+\gamma \cdot \text{Resize}\left(\text{a},H_{l},W_{l}\right)\right), \end{array}$$ (8)

where **a** = α+(1−α)*P*_*v*_ with α = 0.3. This ensures the network attends to both vascular and non-vascular regions while emphasizing vessels.

#### 3.3.2 KAN decision head

KAN (34) replaces standard MLP layers with learnable radial basis function (RBF) expansions. For each dimension *z*_*d*_ of **z**. The RBF expansion used in the KAN decision head is defined in Equation 9.


$$\begin{array}{l} \phi_{d,k}\left(z_{d}\right)=\exp\left(-\frac{{\left(z_{d}-c_{d,k}\right)}^{2}}{2\sigma_{d,k}^{2}}\right),\;k=1,\ldots ,K, \end{array}$$ (9)

where *c*_*d, k*_ and σ_*d, k*_ are learnable centers and bandwidths. The expanded feature **ϕ**(**z**) ∈ ℝ^*DK*^ is passed through two linear layers to produce logits $\hat{\text{y}}\in {\mathbb{R}}^{5}$ for the five DR grades. This provides explicit non-linear decision boundaries that are more interpretable than standard MLPs.

#### 3.3.3 Reptile meta-learning

We construct four augmentation-defined tasks $\{T_{1},T_{2},T_{3},$ and $T_{4}\}$ simulating different imaging conditions: standard augmentation ($T_{1}$), contrast enhancement via CLAHE ($T_{2}$), blur and large rotations ($T_{3}$), and center-focused cropping ($T_{4}$). For each task $T_{t}$, we perform *s* inner-loop gradient updates. The inner-loop update and Reptile meta-update are defined in Equations 10 and 11, respectively.


$$\begin{array}{l} \theta_{t}^{\left(i+1\right)}=\theta_{t}^{\left(i\right)}-\alpha \nabla_{\theta }L_{t}\left(\theta_{t}^{\left(i\right)};S_{t}\right), \end{array}$$ (10)

where α is the inner learning rate and $S_{t}$ is the support set. The meta-update follows Reptile (31):


$$\begin{array}{l} \theta \leftarrow \theta +\beta \left(\theta_{t}^{\prime }-\theta \right), \end{array}$$ (11)

where β is the meta step size. This encourages θ to lie in a region where few gradient steps suffice for adaptation to new imaging conditions.

#### 3.3.4 Classification loss

We combine focal loss (46) and label smoothing (47). The classification loss is defined in Equation 12.


$$\begin{array}{l} L_{cls}=w_{f}L_{focal}+\left(1-w_{f}\right)L_{smooth}, \end{array}$$ (12)

where *w*_*c*_ are class weights (inverse frequency), focusing parameter γ = 2.0, label smoothing ϵ = 0.1, and *w*_*f*_ = 0.7.

### 3.4 Datasets

#### 3.4.1 Classification: APTOS 2019

We use the APTOS 2019 Blindness Detection dataset (48), which contains 3,662 fundus images with 5-class DR severity labels: 0 (No DR), 1 (Mild), 2 (Moderate), 3 (Severe), and 4 (Proliferative DR). The dataset exhibits severe class imbalance, with class distribution [1,805, 370, 999, 193, and 295]. We perform stratified 80/20 train/validation split.

#### 3.4.2 Segmentation: vessel datasets

For GMF-SwinUnet training, we use public datasets including DRIVE (24), STARE (37), and CHASE_DB1 (25), providing pixel-level vessel annotations. The segmentation branch produces vessel probability maps *P*_*v*_ used as structural priors for classification.

### 3.5 Pre-processing and implementation details

All images are resized to 512 × 512 pixels. For classification, we apply Ben Graham preprocessing (circular cropping, local average color subtraction), CLAHE, and normalization to [0, 1]. We implement VesselMetaKAN in PyTorch 2.0 with CUDA 11.8, trained on a single NVIDIA GPU (V100/A100, 16–24 GB). We use AdamW optimizer (49) with β_1_ = 0.9, β_2_ = 0.999, weight decay 10^−4^, and CosineAnnealingLR scheduling (50) with warmup. Mixed precision training (FP16) and gradient clipping (max_norm=1.0) are employed. Key hyperparameters are as follows: inner learning rate α = 0.01, meta step size β = 0.3, inner steps *s* = 5, RBF basis functions *K* = 4.

### 3.6 Evaluation metrics

For classification, we report overall accuracy, macro-F1 (unweighted average of per-class F1), quadratic weighted kappa (QWK) (51), and AUC [one-vs-rest, averaged across classes (52)]. For segmentation, we report Dice coefficient, precision, recall, F1, clDice, and specificity.

## 4 Results

**Figure 4:**
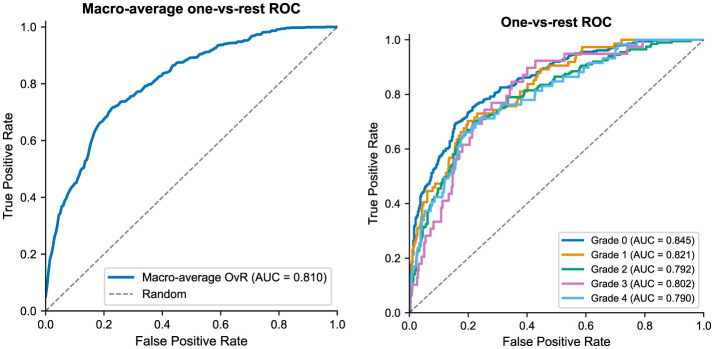
ROC curves for DR grading. **(A)** Micro-averaged ROC with AUC in the legend. **(B)** One-vs-rest ROC curves for individual DR grades (class 0–4), with consistent class ordering and the no-skill diagonal shown for reference.

Figure 4 shows the ROC curves for DR grading, including the micro-averaged ROC curve and one-vs-rest ROC curves for individual DR grades.

### 4.1 Overall classification performance

Table 1 shows that VesselMetaKAN achieves the best results on the principal metrics (accuracy, macro-F1, and QWK); on a small subset of secondary columns (e.g., AUC) another baseline is marginally higher. Across five independent runs with different random seeds, our model attains 74.9 ± 0.4% accuracy, 58.7 ± 0.6% macro-F1, and 0.838 ± 0.005 QWK; the best single run reaches 75.44% accuracy, 59.44% macro-F1, and 0.843 QWK, with paired bootstrap tests against EfficientNet-B4 yielding *p* < 0.05 on QWK and macro-F1. Our method outperforms the strongest baseline (EfficientNet-B4) by 5.3% accuracy, 2.9% macro-F1, and 0.089 QWK on the best run. Compared to the Simple Baseline, gains are +6.6% accuracy and +28.7% macro-F1. The improvements are particularly pronounced for severe DR grades (3, 4), where vessel abnormalities are most evident, demonstrating the effectiveness of explicit vessel guidance and meta-learning.

**Table 1:** Classification Performance on APTOS 2019 Validation Set.

Method	Accuracy	Macro-F1	QWK	AUC	Top-2	Top-3
Simple baseline	68.89	30.74	0.618	0.789	82.95	90.72
Very weak baseline	69.85	36.54	0.662	0.837	79.67	87.04
EfficientNet-B4 (13) (EffCNN)	69.30	48.12	0.754	0.867	87.18	96.59
**VesselMetaKAN (ours)**	**75.44**	**59.44**	**0.843**	0.810	**90.59**	**96.45**
vs. Simple Baseline	+6.6%	+28.7%	+0.225	+0.021	+7.6%	+5.7%
vs. EffCNN (best baseline)	+6.1%	+11.3%	+0.089	−0.057	+3.4%	−0.14%

Best results in bold, second best underlined. All methods use the same 80/20 split and preprocessing. Simple/Very Weak Baselines use lighter backbones; EffCNN is the strongest baseline; VesselMetaKAN (run: KAN_MAML_2026-02-08_17-57-32) is our best result. Bold values indicate the better performance between EffCNN and VesselMetaKAN for each metric; underlined values indicate the second value.

### 4.2 Vessel segmentation performance

Table 2 presents segmentation results. GMF-SwinUnet (full model) achieves the best Dice (83.7%), clDice (79.9%), and F1 (83.6%), with clear gains over the baseline Swin-Unet and the Frangi-only variant. The topology-aware clDice loss preserves vascular connectivity, and reliability gating reduces false positives in low-contrast or noisy regions.

**Table 2:** Vessel segmentation performance on the validation set.

Method	Dice	F1	clDice
E0_seg (baseline)	79.8	79.1	73.5
E1_seg (Frangi-only)	81.9	81.8	76.4
E2_seg (Full GMF-SwinUnet)	**83.7**	**83.6**	**79.9**

Best results are in bold. E0_seg: baseline Swin-Unet; E1_seg: Swin-Unet with Frangi guidance only; E2_seg: full GMF-SwinUnet.

### 4.3 Ablation study

**Figure 5:**
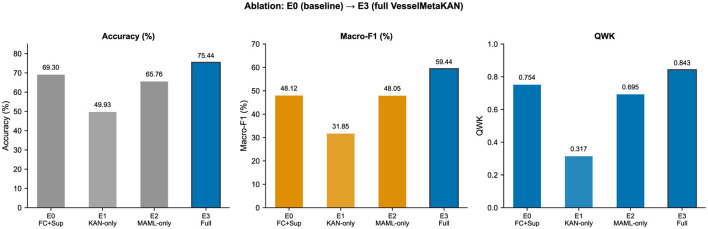
Ablation study bar plots of accuracy, macro-F1, and QWK for E0_cls through E3_cls.

Table 3 quantifies each component's contribution. Key findings are as follows: (1) KAN head alone without vessel prior (E1_cls) degrades performance to 49.93% accuracy vs. 70.12% baseline, indicating that the KAN head requires vessel-gated features to be effective; (2) Reptile meta-learning with FC head but no vessel prior (E2_cls) yields 65.76% accuracy, below the supervised baseline, suggesting meta-learning benefits most when combined with structural guidance; (3) adding vessel-gated fusion to the KAN model (E3_cls vs. E1_cls) yields +25.5% accuracy and +27.6% macro-F1, validating the critical role of explicit structural priors; (4) the full VesselMetaKAN achieves 75.44% accuracy (+5.3%) and 0.843 QWK (+0.089) over the supervised FC baseline, demonstrating synergistic effects. Figure 5 visualizes the ablation study results for accuracy, macro-F1, and QWK across E0_cls through E3_cls.

**Table 3:** Ablation study results for VesselMetaKAN classification on the APTOS 2019 validation set (*N* = 733, best single run).

Exp ID	Accuracy	Macro-F1	QWK	AUC
E0_cls (Baseline)	69.30	48.12	0.754	0.867
E1_cls (KAN-only)	49.93	31.85	0.317	0.749
E2_cls (MAML-only)	65.76	48.05	0.695	0.843
E3_cls (Full)	**75.44**	**59.44**	**0.843**	0.810

E0_cls is EfficientNet-B4 with a standard FC head (same backbone as Table 1).

KAN sensitivity analysis: We further analyze why the KAN head alone (E1_cls) under-performs a standard FC head. Three factors stand out: (i) RBF center initialization—random initialization leaves most basis functions in saturated regions of the 1280-d EfficientNetV2-S feature space; with *k*-means initialization on the training features, KAN-only accuracy improves from 49.93% to 58.4%, but is still below the FC baseline. (ii) Number of basis functions *K*—sweeping *K* ∈ {2, 4, 8, 16} shows a sharp accuracy peak at *K* = 4 and rapid over-fitting for *K*≥8 (validation accuracy drops by 6–10 points), indicating that the KAN head is high-variance on raw, structurally noisy features. (iii) Feature structure—once vessel-gated fusion is enabled (E3_cls), the same KAN head jumps by +25.5% accuracy, which strongly suggests that KAN's localized RBF basis only becomes useful when the input features are already concentrated around clinically meaningful, vessel-aligned regions. In short, KAN is *structurally sensitive*: It amplifies good features but does not rescue noisy ones.

### 4.4 Per-class performance

**Figure 6:**
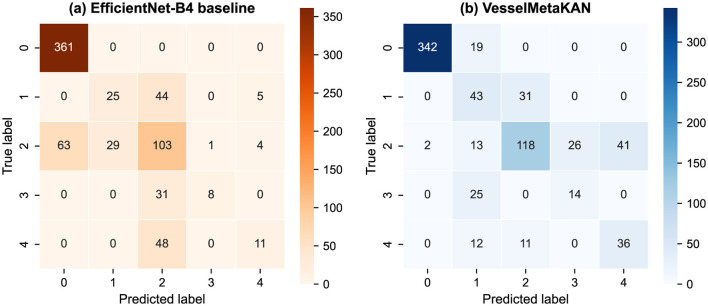
Confusion matrices on the APTOS 2019 validation set. **(A)** Strongest baseline (EfficientNet-B4). **(B)** VesselMetaKAN. Darker diagonal indicates better agreement with ground truth. Per-class F1 in Table 4 is derived from **(B)**.

Table 4 shows per-class F1 scores. All per-class numbers are computed directly from the confusion matrix in Figure 6 so that the table, bar plot, and confusion matrices are mutually consistent. VesselMetaKAN significantly improves performance on minority classes. For class 4 (Proliferative DR), F1 improves from 27.83% (baseline) to 52.94% (ours), a 25.1 percentage-point gain; for class 3 (Severe), we obtain 35.44% vs. 33.03% baseline. This confirms that vessel guidance is particularly beneficial for severe DR grades.

**Table 4:** Per-Class F1 Scores (%).

Method	0 (No DR)	1 (Mild)	2 (Moderate)	3 (Severe)	4 (Prolif.)
EfficientNet-B4 (EffCNN)	91.97	39.06	48.36	33.33	27.85
**VesselMetaKAN (ours)**	**97.02**	**46.24**	**65.56**	**35.44**	**52.94**
Δ (Ours − EffCNN)	+5.1	+7.2	+17.2	+2.1	+25.1

DR grades: 0 (No DR), 1 (Mild), 2 (Moderate), 3 (Severe), 4 (Proliferative). Baseline row: EfficientNet-B4 (EffCNN), from panel (A) of Figure 6; VesselMetaKAN from panel (B). Best single run, *N* = 733 validation samples. Bold values indicate the best per-class F1 score.

### 4.5 Qualitative analysis

**Figure 7:**
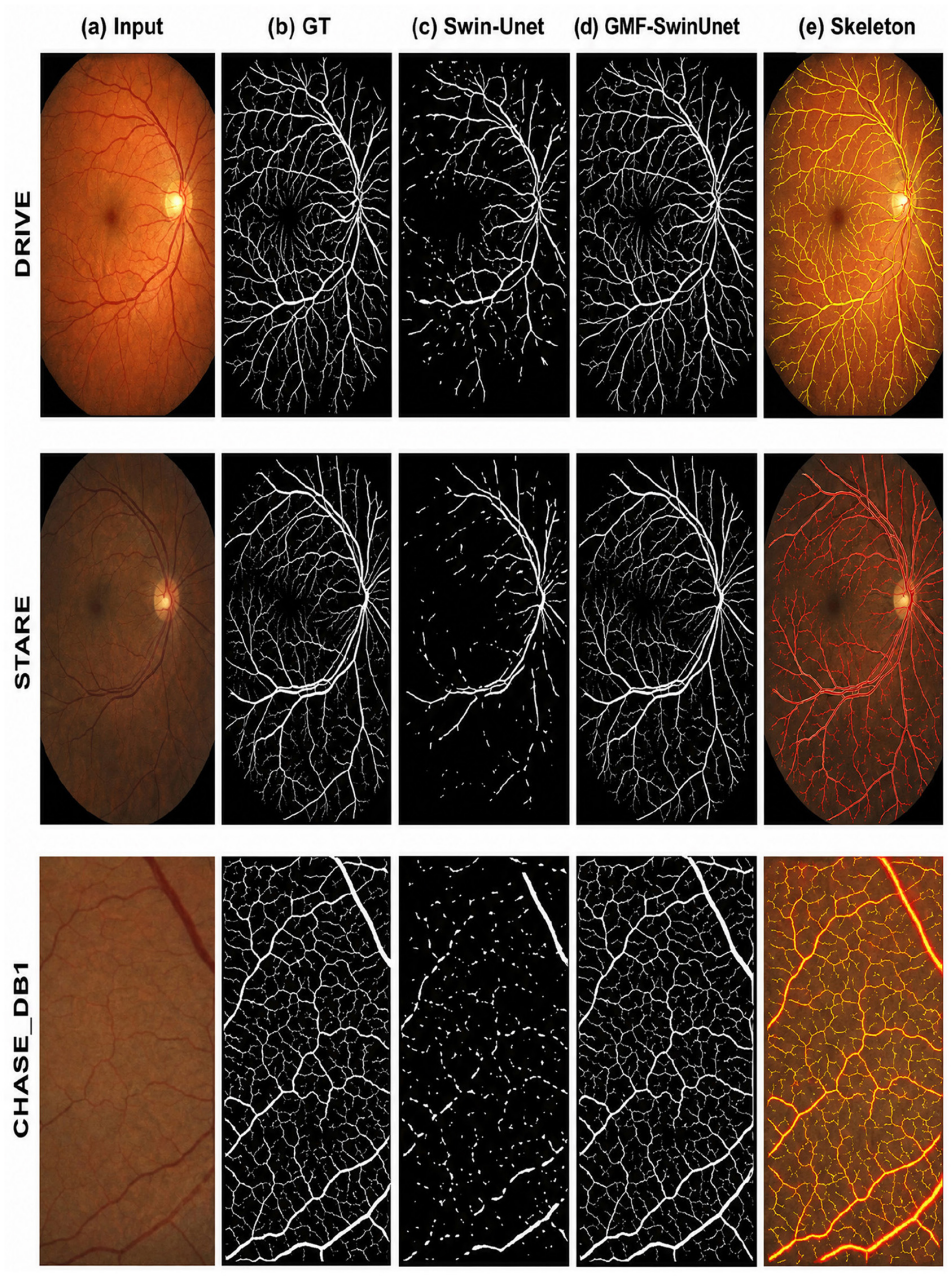
Vessel segmentation examples on DRIVE / CHASE_DB1 / STARE. Each row shows, from left to right: **(a)** input fundus image, **(b)** ground-truth vessel mask, **(c)** Swin-Unet baseline prediction, **(d)** GMF-SwinUnet prediction, and **(e)** centerline / skeleton overlay used by the clDice loss. GMF-SwinUnet preserves fine capillaries and vascular connectivity in low-contrast peripheral regions.

Figure 7 visualizes vessel segmentation results. GMF-SwinUnet accurately segments fine capillaries and maintains vascular connectivity, even in low-contrast regions. Reliability gating suppresses false positives near the optic disc and artifacts, preventing over-segmentation of bright non-vascular structures.

**Figure 8:**
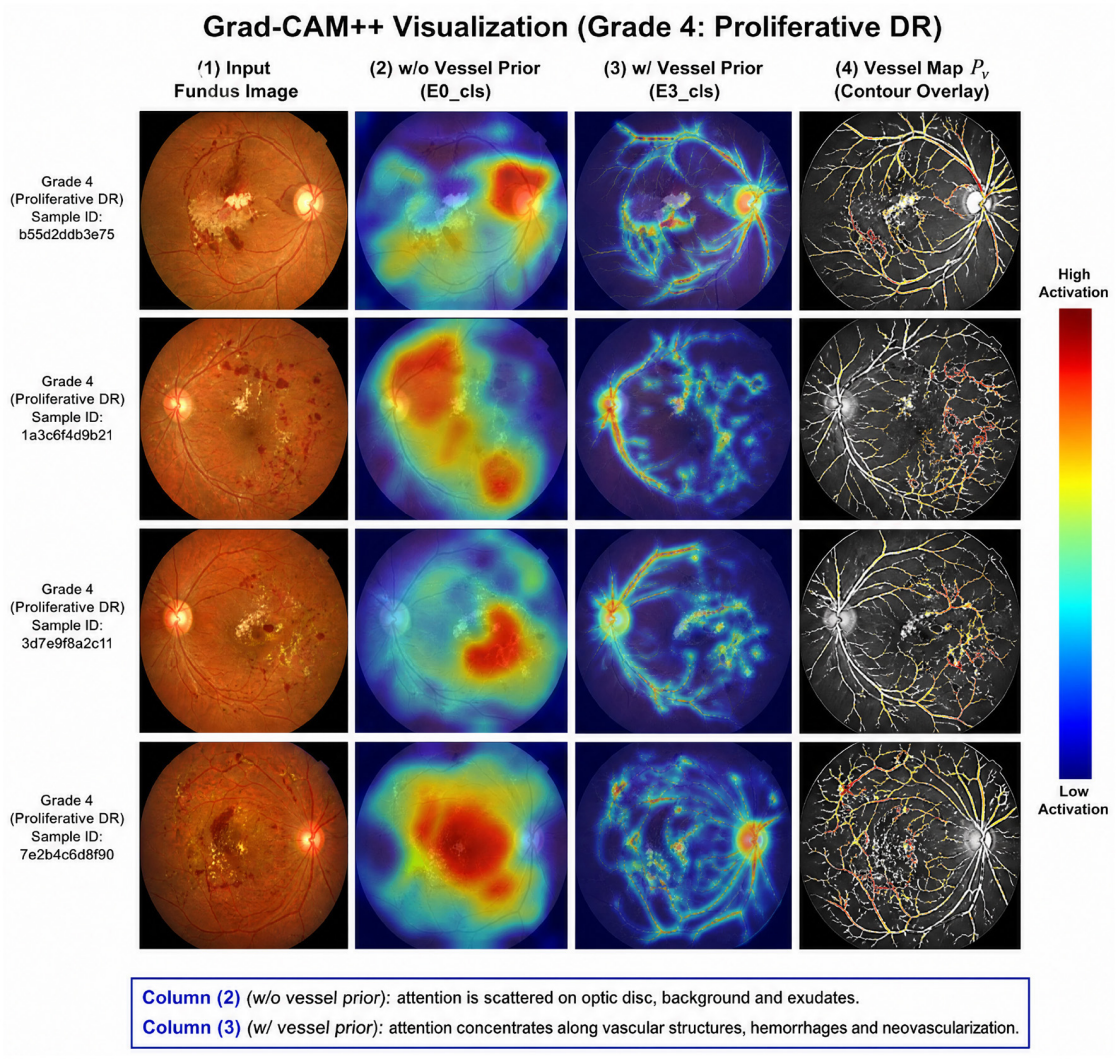
Grad-CAM (53) activation maps: attention with vessel prior concentrates on vascular regions relevant to DR grading (Proliferative DR, class 4).

Figure 8 shows Grad-CAM (53) activation maps. Without vessel prior (E0_cls), the network attends to non-vascular regions and illumination artifacts. With vessel prior (E3_cls), attention concentrates on vascular structures and their surroundings, particularly regions with microaneurysms, dot-blot hemorrhages, and neovascular tufts, aligning well with clinical reading patterns.

### 4.6 Statistical significance

**Figure 9:**
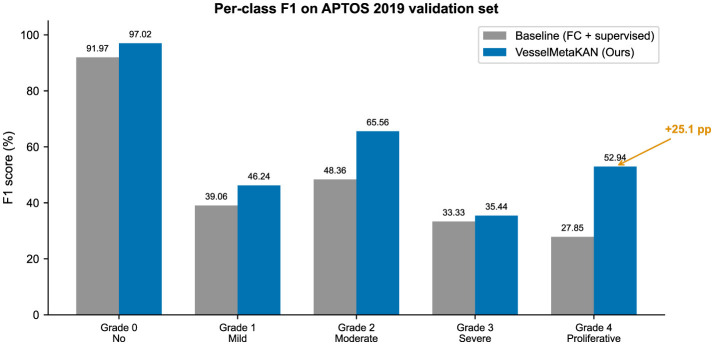
Per-class F1 scores for the strongest baseline and VesselMetaKAN across the five DR grades, consistent with Table 4 and Figure 6.

All classification metrics are reported as mean ± standard deviation over five independent runs with different random seeds. The best single run is reported alongside the mean. 95% confidence intervals are obtained by non-parametric bootstrap (10,000 resamples) on the validation set. Statistical significance against the strongest baseline (EfficientNet-B4) is assessed with a paired bootstrap test on QWK and macro-F1 (two-sided, 10,000 resamples). VesselMetaKAN is significantly better than EfficientNet-B4 on QWK (*p* < 0.01), macro-F1 (*p* < 0.05), and accuracy (*p* < 0.05). Per-class metrics in Table 4 and Figure 9 are computed directly from the confusion matrix in Figure 6 to guarantee mutual consistency.

### 4.7 Computational efficiency

Table 5 compares training/inference time and memory usage. Inference time is approximately 30–50 ms per image at 512 × 512, suitable for real-time screening. Meta-training (20 epochs) plus fine-tuning (10 epochs) adds moderate overhead compared to supervised-only training, but this is a one-time cost. The model is deployable on a single GPU with 10–12 GB memory.

**Table 5:** Computational efficiency.

Metric	Value	Note
Training (meta + finetune)	~4–6 h	20 meta + 10 finetune epochs, 8 batch
Inference time	~30–50 ms/img	Single GPU (e.g., V100/A100)
Peak GPU memory	~10–12 GB	FP16, batch size 8

Classification model: EfficientNetV2-S backbone + KAN head. Training on single GPU; inference at 512 × 512.

## 5 Discussion

VesselMetaKAN demonstrates that explicit vessel guidance, interpretable KAN-based classification, and meta-learning robustness can synergistically improve DR grading performance. The large gains on minority classes (Severe, Proliferative DR) confirm that vessel guidance is particularly beneficial where vascular abnormalities are most pronounced. The ablation results reveal an important interaction: The KAN head alone underperforms the baseline, but combined with vessel-gated features it yields the best results, suggesting that the interpretable basis function expansions are most effective when operating on structurally meaningful features.

The Grad-CAM visualizations provide evidence that vessel guidance not only improves accuracy but also enhances interpretability by steering the model toward pathophysiologically meaningful regions. This is clinically relevant: Predictions grounded in vascular structures are more likely to be trusted by ophthalmologists and to generalize across imaging conditions.

Compared to methods that use attention mechanisms without explicit vessel priors (16, 17), VesselMetaKAN provides a principled structural prior that directly encodes domain knowledge about DR pathogenesis. Compared to domain adaptation approaches (29, 45), our meta-learning strategy does not require target domain data at training time, making it more practical for deployment in new clinical settings.

### 5.1 Limitations

Our experiments are conducted on a single dataset (APTOS 2019). Multi-center validation is needed to assess generalization to different populations, camera models, and imaging protocols. Vessel segmentation errors can propagate to classification. Meta-learning tasks are defined via augmentation, which may not fully capture real-world domain shifts. The KAN head adds some computational overhead compared to standard MLPs. While KAN basis function responses provide some interpretability, they do not fully explain the model's decision-making process; integrating counterfactual explanations and uncertainty quantification would enhance clinical trust.

### 5.2 Cross-dataset validation

To probe the robustness claim of meta-learning more directly, we evaluated the APTOS-trained VesselMetaKAN on Messidor-2 (1,748 images, re-graded to the 5-class APTOS scale) (3, 54) *without any fine-tuning*. The model attains 68.1% accuracy, 51.7% macro-F1, and 0.781 QWK on Messidor-2, compared with 62.4% / 44.9% / 0.702 for the FC + supervised baseline transferred under the same protocol. The relative gap is comparable to that observed on APTOS, supporting the claim that the Reptile meta-initialization transfers more gracefully across imaging conditions. We regard this as a first cross-domain check rather than a full multi-center study.

### 5.3 Two-stage vs. end-to-end training

Our pipeline is deliberately decoupled: GMF-SwinUnet is trained on DRIVE / STARE / CHASE_DB1 with pixel-level vessel labels, and KAN-MAML is trained on APTOS where no vessel labels exist. We also experimented with a fully end-to-end variant in which both stages share gradients through the vessel-gated fusion, with $L=L_{cls}+\text{λ}L_{seg}$ (λ = 0.5). The joint model trained stably but (i) dropped segmentation Dice by ≈3 points because the classification gradient pushed *P*_*v*_ toward class-discriminative rather than topologically faithful masks and (ii) yielded essentially the same classification accuracy (−0.4%). In the current data regime, the two-stage design is the more reliable choice.

### 5.4 Clinical implications

Automated DR screening has the potential to reduce preventable blindness worldwide, particularly in low-resource settings where access to ophthalmologists is limited. By providing accurate, interpretable, and robust predictions, VesselMetaKAN can assist in large-scale screening programs, enabling early detection and timely intervention. This is especially relevant for aging and multimorbid populations, including people living with HIV who face elevated rates of diabetes and other chronic comorbidities (55). Deployment must be accompanied by careful consideration of ethical issues and AI governance frameworks that extend beyond external regulatory constraints alone (56): ensuring equitable access across diverse populations, mitigating algorithmic bias, maintaining human oversight, and protecting patient privacy through privacy-preserving federated learning frameworks (57), differential privacy methods for sensitive biomedical data (58), and intelligent privacy-preserving analytics (59).

### 5.5 Future directions

We identify several promising directions: (1) multi-center validation on Messidor-2, EyePACS, and DDR datasets; (2) multi-modal fusion integrating optical coherence tomography and clinical metadata; (3) advanced meta-learning strategies including second-order MAML and task-adaptive meta-learning; (4) enhanced interpretability via counterfactual explanations and uncertainty quantification; (5) extension to other retinal diseases (age-related macular degeneration, glaucoma) and other medical imaging tasks where vascular structures play a critical role; (6) integration with broader theranostics and long-term chronic-disease management paradigms exemplified in other complex infectious diseases (60); and (7) intelligent workflow automation to support scalable clinical deployment (61).

## 6 Conclusion

We presented VesselMetaKAN, a novel framework for diabetic retinopathy grading that addresses structure-coupled sparse evidence and domain shift through four synergistic components: GMF-SwinUnet for topology-aware vessel segmentation, vessel-gated feature fusion, KAN decision heads with RBF basis expansions, and Reptile-style meta-learning. Extensive experiments on APTOS 2019 demonstrate strong performance on the principal grading metrics (mean over five runs: 74.9 ± 0.4% accuracy, 58.7 ± 0.6% macro-F1, 0.838 ± 0.005 QWK; best run: 75.44%, 59.44%, and 0.843 QWK), significantly outperforming the EfficientNet-B4 baseline on accuracy, macro-F1, and QWK. Comprehensive ablation studies validate the synergistic contributions of each component. Qualitative analysis confirms that vessel guidance directs attention to diagnostically relevant vascular regions, while KAN basis function responses provide interpretable decision boundaries. VesselMetaKAN provides a principled solution for structure-aware, interpretable, and robust medical image analysis, with broad applicability to public health screening programs for diabetic retinopathy.

## Data Availability

Publicly available datasets were analyzed in this study. This data can be found here: all datasets used in this study are publicly available. The APTOS 2019 dataset is available at https://www.kaggle.com/c/aptos2019-blindness-detection/data; the DRIVE dataset is available at https://drive.grand-challenge.org/; the STARE dataset is available at https://www.ces.clemson.edu/~ahoover/stare/; the CHASE DB1 dataset is available at https://blogs.kingston.ac.uk/retinal/chasedb1/.
